# *Allium stipitatum* Extract Exhibits In Vivo Antibacterial Activity against Methicillin-Resistant* Staphylococcus aureus* and Accelerates Burn Wound Healing in a Full-Thickness Murine Burn Model

**DOI:** 10.1155/2017/1914732

**Published:** 2017-02-22

**Authors:** Arunkumar Karunanidhi, Ehsanollah Ghaznavi-Rad, Jayakayatri Jeevajothi Nathan, Yusuf Abba, Alex van Belkum, Vasanthakumari Neela

**Affiliations:** ^1^Department of Medical Microbiology and Parasitology, Faculty of Medicine and Health Sciences, Universiti Putra Malaysia, 43400 Serdang, Selangor, Darul Ehsan, Malaysia; ^2^Department of Microbiology & Immunology, Faculty of Medicine, Arak University of Medical Sciences, Basij Square, Arak 38481-7-6941, Iran; ^3^School of Medicine, Taylor's University, Lakeside Campus, No. 1, Jalan Taylor's, Subang Jaya, 47500 Selangor, Darul Ehsan, Malaysia; ^4^Department of Pathology and Microbiology, Faculty of Veterinary Medicine, Universiti Putra Malaysia, 43400 Serdang, Selangor, Darul Ehsan, Malaysia; ^5^BioMérieux, Scientific Office, La Balme les Grottes, France

## Abstract

The in vivo antibacterial and burn wound healing potency of Persian shallot bulbs (*Allium stipitatum*) were explored in a mice burn model infected with methicillin-resistant* Staphylococcus aureus* (MRSA). Hexane (ASHE) and dichloromethane (ASDE) extracts were tested. Female BALB/c mice were inflicted with third-degree thermal injury followed by infection with MRSA. ASHE and ASDE formulated with simple ointment base (SOB) at concentrations of 1%, 2%, and 5% (w/w) were topically applied to burn wounds twice a day for 20 days. Silver sulfadiazine (1%) served as drug positive control. Microbiological analysis was carried out on 1, 2, 3, 4, and 5 days postwounding (dpw) and histopathological analysis at the end of the experiment (20 dpw). Both ointments demonstrated strong antibacterial activity with complete elimination of MRSA at 48–72 h after infection. The rate of wound contraction was higher (95–100%) in mice groups treated with ASHE and ASDE ointments after 15 dpw. Histological analysis revealed significant increase (*p* < 0.05) in epithelialization and collagenation in treated groups. The ASHE and ASDE were found to be relatively noncytotoxic and safe to Vero cell line (383.4 *μ*g mL^−1^; 390.6 *μ*g mL^−1^), suggesting the extracts as safe topical antibacterial as well as promising alternatives in managing thermal injuries.

## 1. Introduction

Thermal injuries are one of the most common and confounding forms of serious injuries worldwide. The contingency of risks due to cutaneous and systemic infections due to burns is higher in patients suffering from stern thermal wounds. Despite the advances in burn wound care and treatments, the incidences and death rates are increasing to a large extent, substantially due to ensuing microbial infections in burn individuals [1]. The estimated incidences of thermal injuries account for ~265,000 deaths each year. Majority of the incidences are reported from low- and middle-income countries and nearly 50% incidences were reported from the Southeast Asia region [2]. Burn wound infections due to MDR pathogens further complicate the healing process and require immediate medical attention with precise antibiotic treatment. Invasive burn wound infections by* A. baumannii*, coagulase-negative staphylococci,* Enterococcus* spp.,* P. aeruginosa*,* S. maltophilia*, VRE, and* Candida albicans* have been frequently reported in burn units [3–8].

Wounds contaminated with MRSA are a principal cause of morbidity and mortality in hospitalized patients with thermal injuries and surgical and skin and soft tissue infections (SSTIs) [9]. The primary catastrophes of burn wounds contaminated with MRSA include the spread of the pathogen to other parts of the body leading to delayed wound healing in the infected site [10], followed by systemic infection, sepsis, organ failures, and deaths [1]. Secondary risk includes the possibility of increased transmission of the pathogen from burn patients to nonburn patients [11].

Silver sulfadiazine (SSD) is one of the most commonly employed antimicrobial agents for partial-thickness and full-thickness burns. Although SSD is active against a broad spectrum of microorganisms, disadvantages like poor penetration to eschars and the ability to cause leukopenia further limit SSD as the most suitable therapeutic option for burn wounds [12]. The treatment of burn wounds in clinical settings continues to be a clinical challenge and an economic burden [13], with limited therapeutic options to help prevent wound expansion, and therefore new approaches are required. In particular, plant extracts are considered remarkable and promote wound healing through expression and release of growth factors and cytokines, or by accelerating blood clotting, collagenation, and epithelialization [14, 15].

Persian shallot (*A. stipitatum*) has wound healing properties [16] and has been widely used in Iran and its neighboring countries such as Tajikistan and Uzbekistan as an important ethnomedicinal plant [17]. The distinctive nature of this plant from other shallots like* A. ascalonicum* Linn. (common shallot), makes it an important medicinal plant with unexplored medicinal properties. The bulbs of* A. stipitatum* have been reported to have wound healing [16], antispasmodic [18], and anti-inflammatory properties [19]. Recently, hydromethanolic extract of Persian shallot was shown to be effective against a panel of clinically important pathogens such as MRSA, methicillin-sensitive* S. aureus* (MSSA),* S. epidermidis*,* Streptococcus pneumoniae*,* Escherichia coli*,* Salmonella typhimurium*,* Proteus mirabilis*, and* Klebsiella pneumoniae *[20]. However, the knowledge of this medicinal plant in the treatment of thermal injuries is yet to be explored. In our preliminary screening, we employed sequential extraction method using various solvents from nonpolar to polar, starting from hexane, ethyl acetate, dichloromethane, methanol, and water. We subjected all the extracts to the antibacterial activity against MRSA and found promising activity in hexane (ASHE) and dichloromethane (ASDE) extracts compared to others. Therefore, the present study was aimed to investigate the wound healing activity of ASHE and ASDE formulated ointment in a MRSA induced murine burn model.

## 2. Materials and Methods

### 2.1. Plant Material and Preparation of Extracts

Fresh bulbs of Persian shallot (*Allium stipitatum*) were collected at 1750 m above sea level in Arak, Markazi Province of Iran (33°05′N, 49°42′E).* A. stipitatum* was authenticated by taxonomist Dr. Mitra Noori and the voucher specimen (CMN 10, 02 May, 2007) was deposited at the Department of Biology, Faculty of Science, Arak University, Iran. Plant materials were washed with water, sliced to pieces, and dried completely under shade for 2-3 weeks. Five kilograms of dried Persian shallot bulbs was ground into fine powder and extracted with hexane for 72 h using maceration and subsequently with dichloromethane. The extracts were filtered through Whatman No. 1 paper to remove solid plant materials and the filtrates were dried under vacuum (BÜCHI Rotavapor R-200, Flawil, Switzerland) at 40°C. Upon filtration and solvent volatilization, the hexane and dichloromethane extracts (ASHE and ASDE) of* A. stipitatum* yielded 221 g (4.42%) and 164 g (3.28%) of residue, respectively. The concentrates were transferred to glass scintillation vials (Wheaton Brand, USA) and used for further assays.

### 2.2. Chemicals

Brain Heart Infusion (BHI) broth, mannitol salt agar, trypticase soy broth (TSB), and Mueller-Hinton agar were purchased from Becton-Dickinson, Cockeysville, MD. Balb/c mice were supplied by KRK Seri Enterprise, Selangor, Malaysia. Vero cell line (African green monkey cells) was purchased from the American Tissue Culture Collection (Manassas, VA, USA). Silver sulfadiazine (T.O. Chemicals, Bangkok, Thailand), xylazine (CEVA Sante Animale, Maassluis, The Netherlands), ketamine 62.5 mg/kg (Aescoket; Aesculaap, Boxtel, The Netherlands), and cylindrical brass heating device [200 mm (*l*) × 10 mm (*b*) × 10 mm (*h*)] were purchased from commercial vendors. Simple ointment base (SOB) was kindly given as a gift by Dr. Senthilrajan from the Department of Pharmacy, International Medical University, Malaysia.

### 2.3. Gas Chromatography-Mass Spectrometry (GC-MS) Analysis

The chemical constituents of ASHE and ASDE were analyzed by an Agilent 7890A Gas chromatograph system with an Agilent 5975C inert mass selective detector (MSD). The machine was equipped with a HP-5MS capillary column (30 × 0.25 mm, film thickness 0.25 *μ*m). For GC-MS detection, an electron ionization energy of 70 eV was used. Helium at a constant flow rate of 1.0 mL min^−1^ was employed as a carrier gas. Injector and MS transfer line temperature were set at 220°C and 290°C, respectively. Oven temperature was programmed as follows: 60°C for 15 min, increased to 150°C at 3°C min^−1^, 10 min hold at 150°C, and then increased up to 230°C at 3°C min^−1^ and 10 min hold at 230°C. Diluted samples (1/100, v/v, in dichloromethane) of 1.0 *μ*L were injected manually in splitless mode and the relative percentages of ASHE and ASDE constituents were expressed as percentages by peak area normalization. Qualitative identification of the different constituents was performed by comparison of their relative retention times and mass spectra with those of Wiley and National Institute of Standards and Technology (NIST) Mass spectral libraries (NIST/EPA/EPA-2008 version) and literature data. 

### 2.4. Antibacterial Activities

#### 2.4.1. Test Microorganism

Methicillin-resistant* S. aureus* (MRSA) ATCC 43300 was used as the representative burn pathogen. MRSA culture was stored in brain heart (BHI) broth supplemented with 20% glycerol at −80°C. For in vitro antimicrobial assays and assessment and animal infection experiments, MRSA culture from frozen stocks was grown overnight in TSB at 37°C in a shaking incubator (180 rpm). Optical density (OD) of the bacterial culture was adjusted to 0.5 McFarland standard at 600 nm using a Biophotometer (Eppendorf Biophotometer* plus*, Hamburg, Germany).

#### 2.4.2. Disk Diffusion Assay

The antibacterial activity of ASHE and ASDE was assessed by disk diffusion method following the guidelines recommended by Clinical Laboratory Standards Institute [21]. Standardized overnight bacterial cultures at a concentration of 0.5 McFarland (1 × 10^8^ CFU mL^−1^) was spread onto Mueller-Hinton agar (MHA) plates by lawn culture. For disk diffusion assays, ASHE and ASDE were prepared freshly at a concentration of 10 mg mL^−1^ in 10% DMSO. Sterile antibiotic assay filter paper discs of 6 mm diameter were placed on MHA plates and 20 *μ*L (10 mg mL^−1^, corresponds to 200 *μ*g extract) of ASHE or ASDE was loaded onto the filter paper discs. Vancomycin antibiotic was used as positive control, while filter paper disc loaded with 20 *μ*L of DMSO (10%) was included as a negative control (diluent control). The plates were incubated at 37°C and the inhibition zones were measured after 24 h.

#### 2.4.3. Determination of Minimum Inhibitory Concentration (MIC) and Minimum Bactericidal Concentration (MBC)

MIC is the lowest concentration of an antimicrobial agent that inhibits the visible growth of a microorganism after overnight incubation (Martin et al., 2003), while the lowest amount of an antimicrobial agent that kills all the bacterial cells was defined as MBC. The MICs of ASHE and ASDE for MRSA were determined by broth microdilution method following the CLSI guidelines [21]. Briefly, 100 *μ*L of Mueller-Hinton broth (MHB) was pipetted into each well of a clear and sterile, flat-bottomed 96-well microplate. ASHE and ASDE were prepared freshly at a concentration of 8192 mg mL^−1^ in 10% DMSO. Successive 2-fold serial dilutions were performed in 0.1 mL of MHB to give a final concentration ranging from 8192 to 1.0 *μ*g mL^−1^ of ASHE and ASDE in each well. Bacterial inoculum containing ~10^5^ bacterial cells (100 *μ*L) was inoculated to all wells except the broth control wells and the plate was incubated at 37°C for 24 h. After 24 h, 30 *μ*L of resazurin (0.01%) was added to the plates and further incubated for 6–8 h. A color change from blue to pink indicated the bacterial growth in the wells. The MBC was determined by spread plating 100 *μ*L of the broth from clear wells (blue color) onto MHA plates followed by a 24 h incubation at 37°C.

### 2.5. Preparation of Formulations

Hexane (ASHE) and dichloromethane (ASDE) extracts of* A. stipitatum* were formulated with simple ointment base (SOB) BP:1980 as a vehicle [22]. Briefly, simple ointment base was prepared by mixing the ingredients (wool fat 5 g, hard paraffin 5 g, cetostearyl alcohol 5 g, and white soft paraffin 85 g) according to British Pharmacopoeia (1980) in a beaker placed in a 65°C water bath. A homogeneous mixture was obtained upon constant stirring and the mixture was cooled and homogenized at 1500 rpm for 10–15 min. Two individual topical formulations, each containing ointment base (50 g) plus ASHE (0.5 g, 1.0 g, and 2.5 g) and ointment base (50 g) plus ASDE (0.5 g, 1.0 g, and 2.5 g), were prepared. A homogenous mixture of the ointment base mixed with extract was achieved by appropriate stirring. The final concentrations of each formulation contained 0.5 g, 1.0 g, and 2.5 g of extract with 50 g of ointment base yielding 1%, 2%, and 5% w/w of active extract.

### 2.6. Ethical Consideration

This study was conducted at the Animal Experimental Unit, Faculty of Medicine and Health Sciences, Universiti Putra Malaysia (UPM), and ethical approval was granted by the University Animal Ethics Committee, Universiti Putra Malaysia (Approval Number UPM/FPSK/PADS/BR-UUH/00424). Animals were humanely handled and euthanized at stipulated dates during the experimental period, using a CO_2_ chamber after anesthesia with Ketamine + Xylazine (50 mg kg^−1^ + 10 mg kg^−1^).

### 2.7. Experimental Animals

One hundred and thirty-five BALB/c female mice (17–21 g; 7-8 weeks of age) were included in this study. The mice were allowed to acclimatize in the animal facility for 10 days with free access to food and water. Animals were grouped into 2 categories [infection-free groups (9 groups; *n* = 45) and infected with MRSA (9 groups; *n* = 90)] comprising 18 groups. Mice groupings were as follows: Group 1, burn wound; Group 2, burn + SOB; Group 3, burn + MRSA; Group 4, burn + MRSA + SOB; Group 5, burn + SSD (1%); Group 6, burn + MRSA + SSD (1%); Group 7, burn + MRSA + ASHE (1%); Group 8, burn + MRSA + ASHE (2%); Group 9, burn + MRSA + ASHE (5%); Group 10, burn + ASHE (1%); Group 11, burn + ASHE (2%); Group 12, burn + ASHE (5%); Group 13, burn + MRSA + ASDE (1%); Group 14, burn + MRSA + ASDE (2%); Group 15, burn + MRSA + ASDE (5%); Group 16, burn + ASDE (1%); Group 17, burn + ASDE (2%); Group 18, burn + ASDE (5%). Infection-free groups contained 5 mice per group, while burn pathogen infected groups consisted of 10 mice per group. At day 5, day 10, day 15, and day 20 of infection and treatment, two mice per group were euthanized using a CO_2_ chamber after anesthesia with Ketamine + Xylazine (50 mg kg^−1^ + 10 mg kg^−1^) for microbiological and histological evaluations.

### 2.8. Burn Injury Procedure

Thermal injury procedure was introduced according to the method described previously [23]. Briefly, the dorsal surface of the mice was shaved (2 × 3 cm) one day prior to the experiment. Mice were anaesthetized intraperitoneally with ketamine hydrochloride (50 mg/kg b.w.) and xylazine (10 mg/kg b.w.). All mice received an even full-thickness burn of 1 × 1 cm (third-degree) on the shaved area using a heated brass for 5 seconds [24]. The mice were resuscitated with 1 mL of physiological saline (0.85% NaCl) intraperitoneally before inoculation of bacterial culture on the burn wound surfaces.

### 2.9. Challenge and Treatment

Mice were challenged with MRSA ATCC 43300 according to the method described earlier [25]. MRSA suspension was prepared from overnight cultures grown in nutrient broth at 37°C with a final concentration of ~10^5^ to 10^6^ CFU mL^−1^. Immediately after the wound was created, mice were inoculated with 10 *μ*L of MRSA culture evenly applied topically on the burn site after a waiting period of 30 minutes [26]. The bacterial inoculum over the burn wound was allowed to dry and the animals were returned to their respective cages. Twenty-four hours postinfection (hpi), 20 mg of ASHE and ASDE ointments at different concentrations (1%, 2%, and 5%) were applied on the skin burns, twice a day for a period of 20 days. Uninfected mice that were either untreated or treated with ASHE/ASDE/SSD served as additional controls. Groups receiving SOB served as negative control.

### 2.10. Microbiological Analysis

Wound tissues (1 × 1 cm) were excised after euthanasia using sterile surgical scissors and subjected to microbiological analysis after 24 hpi and treatment. Tissue samples were homogenized in 1 mL of sterile PBS, and 10-fold serially diluted bacterial colonies were enumerated by plating on MSA. The results were normalized and expressed in log_10_ CFU bacterial load present in one gram of tissue biopsy sample [25].

### 2.11. Evaluation of Wound Healing Activity and Rate of Wound Contraction

The progressive healing of the wound size was evaluated by tracing the wound after every 5-day interval (for 20 consecutive days) using transparent paper and a pencil, and the recorded wound area was measured graphically [23]. Wound contraction rates were measured as a percentage reduction in wound size after every 5-day interval. Wound contraction was calculated as follows:

(1) Percentage of wound  contraction=Original wound area−Unhealed areaOriginal wound area×100%.

### 2.12. Histopathological Analysis

For histopathological analysis, one mouse was euthanized from each group on the 20th day after wounding. Tissue samples (2 × 3 mm) were excised, fixed in buffered formalin (10%), and dehydrated with alcohol and finally embedded in paraffin wax into blocks. Thin sections of tissue samples (5*μ*m) were stained with hematoxylin and eosin (H&E) for evaluation of pathological changes. The slides were examined under a Leica DFC295 light microscope and lesions in the skin sections were evaluated in terms of congestion, edema, polymorphonuclear leukocytes (PMN) infiltration, mononuclear leukocytes (MNC) infiltration, ulceration, degeneration and necrosis, neovascularization, fibroblast proliferation, and epithelialization. Severity of burns and signs of wound healing were determined in one unit of microscopic focus field, equivalent to 2 × 2 mm^2^ area of the tissue as described by [27] with modifications (0: no lesion distribution; 1: mild distribution; 2: moderate distribution; and 3: marked distribution).

### 2.13. Cytotoxicity Assay on Vero Cell Lines

The cytotoxicity of ASHE and ASDE was determined on Vero cell line using the 3-(4,5-dimethylthiazol-2-yl)-2,5-diphenyltetrazolium bromide (MTT) assay [28]. Cell lines were maintained in RPMI-1640 media (Sigma, USA), enriched with heat-activated fetal bovine serum FBS (10%), 1% Penicillin-Streptomycin, and amphotericin B (Life Technologies). Cells were grown in 75 cm^3^ tissue culture flasks in a humidified atmosphere containing 5% CO_2_ at 37°C. Confluent monolayer cells were washed with PBS, trypsinized, and subcultured in a 96-well microtitre plate at a concentration of 6.0 × 10^4^ cells per well. Following 24 h of preconditioning, culture medium was aspirated and cells were treated with twofold dilutions (10000, 5000, 2500, 1250, 625, 312.5, 156.25, 78.13, 39.03, and 19.54 *μ*g mL^−1^) of ASHE and ASDE for 24 h diluted in RPMI-1640 with 2% FBS. Subsequently, 20 *μ*L of MTT dye (5 mg/mL in PBS) was added to the wells and incubated at 37°C for an additional 3 h. One hundred and fifty microliters of DMSO was added to the wells and incubated for 30 minutes. The cell viability indices were calculated by measuring the optical density (OD) of the color produced by MTT dye reduction at 570 nm using the following formula:

(2) $$\begin{array}{c} \%\text{ cytotoxicity}=\left(\;\frac{A_{570}\text{ of treated cells}}{A_{570}\text{ of control cells}}\;\right)\times 100\%. \end{array}$$

### 2.14. Statistical Analysis

Statistical analysis was performed using GraphPad Prism software (GraphPad, San Diego, CA, USA) by one-way analysis of variance (ANOVA) with Dunnett's multiple comparison after test. Values are expressed as mean ± SE of 5 animals per group. ^*∗*^*p* < 0.05, ^*∗∗*^*p* < 0.01, and ^*∗∗∗*^*p* < 0.001 compared with the control.

## 3. Results

### 3.1. Chemical Constituents of ASHE and ASDE Analyzed by GC-MS

The results of the phytochemical constituents of ASHE and ASDE are shown in Tables 1(a) and 1(b). ASHE and ASDE revealed a strong presence of essential fatty acids, decanoic acid, 9,12-octadecadienoic acid, monoterpenoids, saturated/unsaturated fatty acids (linolenic acid and linoleic acid), organosulfur compounds, aromatic alcohol, synthetic intermediates, organic ester fatty acids, and aromatic hydrocarbons. Both extracts contained high concentrations of *γ*-hexalactone (19.37%), followed by 9,12-octadecanoic acid (19.17%) and hexadecanoic acid (19.37%). Other organic compounds were found in traces (1–10%). Similarly, both extracts contained low percentages of organosulfur compounds like methanesulfonamide (5.23%), 2-pyridinethione (3.87%), 2-mercaptopyridine (1.68%), thiosulfuric acid (1.52%), and cysteaminesulfonic acid (0.25%).

### 3.2. Antibacterial Activities of ASHE and ASDE (Zone of Inhibition, MICs and MBCs)

**Figure 1:**
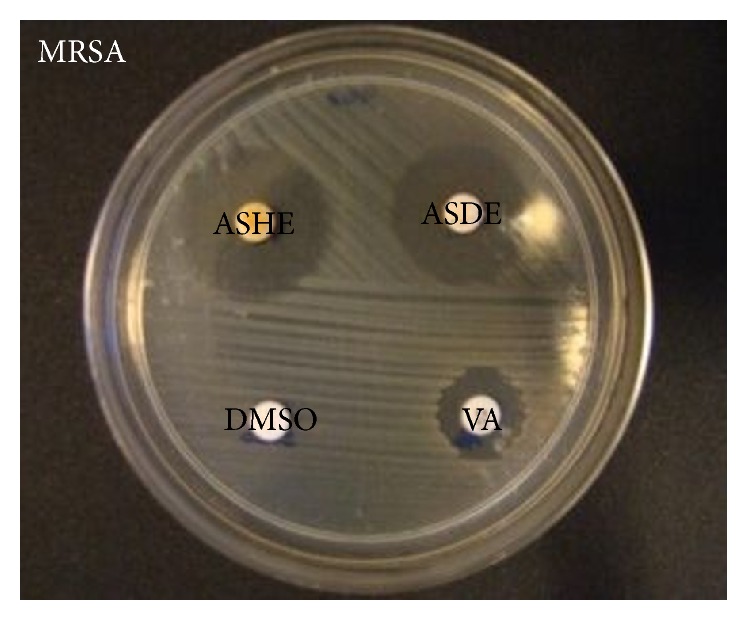
Antibacterial activity of ASHE (200 *μ*g/disc) and ASDE (200 *μ*g/disc) in disc diffusion assay plate showing the halos against MRSA ATCC 43300.

Both ASHE and ASDE showed promising antibacterial activity against MRSA and the inhibition zones for ASHE and ASDE were 27 and 23 mm, respectively (Figure 1; Table 2). The MICs of ASHE and ASDE ranged from 32 to 64 *μ*g mL^−1^ and the MBCs of both extracts were 128 *μ*g mL^−1^ (Table 2).

### 3.3. In Vivo Antibacterial Activity of ASHE and ASDE on Burn Pathogens

**Figure 2:**
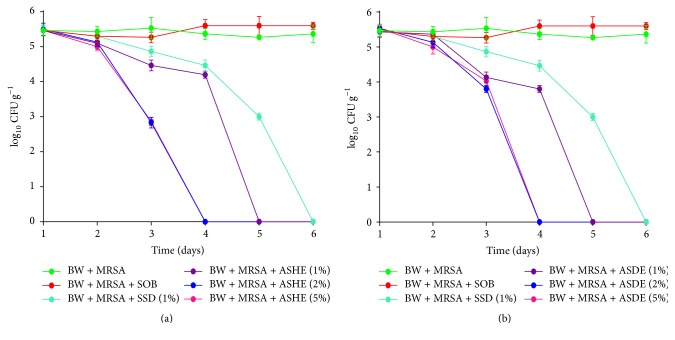
Effect of (a) ASHE (1%, 2% , and 5%) and (b) ASDE (1%, 2%,  and 5%) treatments versus SSD (1%) on the density of MRSA in burn wound infection.

The in vivo antibacterial activity of ASHE and ASDE against MRSA was determined by measurements of tissue bacterial burden. Topical application of ASHE and ASDE formulated ointments suppressed the bacterial load when compared to the control groups (Figures 2(a) and 2(b)). ASHE and ASDE exhibited antibacterial activities in a concentration dependent manner, where 2% and 5% extracts exhibit strong antibacterial activity compared to 1%. ASHE and ASDE at 5% concentration showed a significant 2-3 log_10_ reduction at earlier time points; however complete elimination of MRSA was observed after 72 hpi (0 log_10_ CFU g^−1^). Meanwhile, 1% ASHE/ASDE showed a slower bactericidal efficacy at 96 hpi with ~0.5 log_10_ reduction in CFU g^−1^. SSD (1%) was also found to be effective against MRSA with a complete bactericidal activity for MRSA observed at 96 hpi (with ~0.53 log_10_ reduction in CFU g^−1^). No reduction in bacterial load was observed in additional controls, that is, mice with burn + infection and burn + infection + SOB (Figures 2(a) and 2(b)).

### 3.4. The Rate of Wound Healing in Mice Increased with ASHE and ASDE Treatments

**Figure 3:**
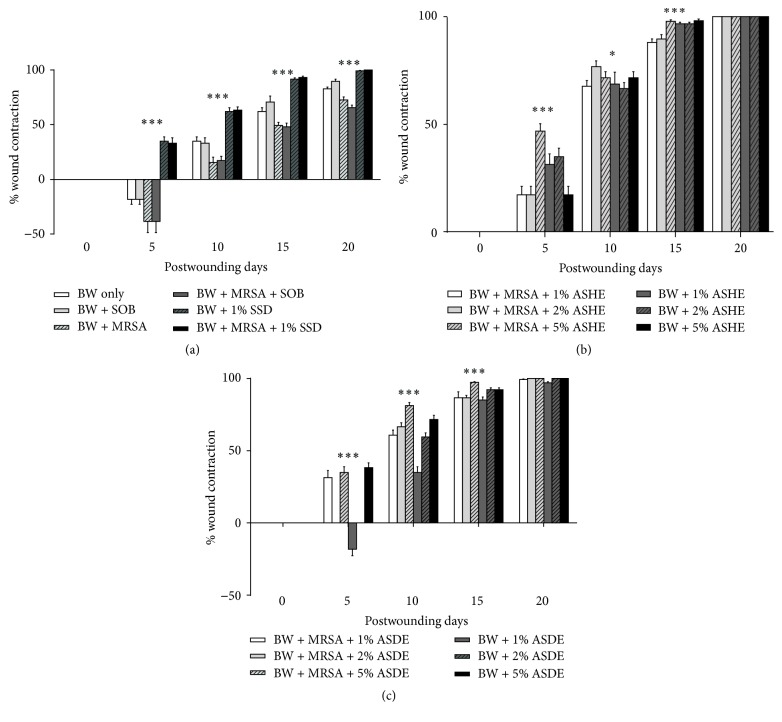
(a) Effect of positive (SSD), negative (BW only), and additional controls (SOB) on burn wound contraction in MRSA infected and uninfected control groups. Values are expressed as mean ± SD of five animals in each group. *∗∗∗* represents *p* < 0.001 compared with control (one-way ANOVA with Dunnett's multiple comparison). (b) Effect of ASHE (1%, 2%, and 5%) ointment formulation on burn wound contraction in MRSA infected and uninfected control groups. Values are expressed as mean ± SD of five animals in each group. ^*∗*^*p* < 0.05 and ^*∗∗∗*^*p* < 0.001 compared with control (one-way ANOVA with Dunnett's multiple comparison). (c) Effect of ASDE (1%, 2%, and 5%) ointment formulation on burn wound contraction in MRSA infected and uninfected control groups. Values are expressed as mean ± SD of five animals in each group. *∗∗∗* represents *p* < 0.001 compared with control (one-way ANOVA with Dunnett's multiple comparison).

The rate of wound healing in terms of percentage of wound contraction was significantly higher (*p* < 0.05) in mice groups that received ASHE and ASDE treatments. ASHE and ASDE at 5% concentrations were found to be effective in healing burn wounds as early as 15 dpw (Figures 3(b) and 3(c); Figures S1b and c in Supplementary Material, available online at https://doi.org/10.1155/2017/1914732), as compared to groups treated with 1% SSD, which showed ~90% wound contraction after 20 dpw (Figure 3(a) and Figure S1a). Mice burns (uninfected and MRSA infected) treated with ASHE and ASDE (2% and 5%) resulted in 100% wound contraction on day 15, whereas 1% ASHE and ASDE treatments resulted in ~90% wound contraction after 15 days (Figure 3(b)). However, the control wounds showed only less than 50% of wound contraction even after 20 dpw in untreated and SOB treated groups.

### 3.5. Histological Analyses

**Figure 4:**
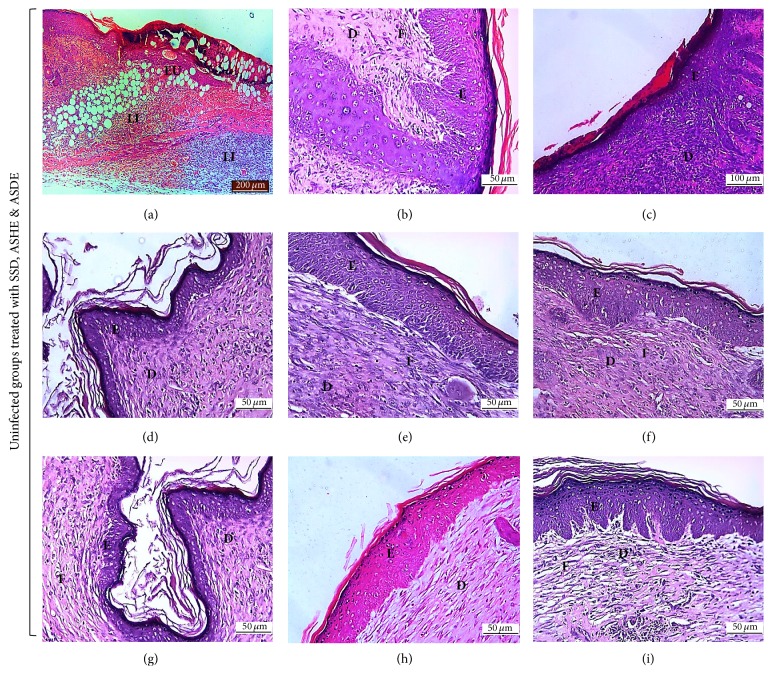
Representative photomicrographs of burn wound (uninfected treated) healing in different treatment groups. Skin sections showing an area of (a) mice with burn wound (normal control) showing epidermal ulceration (EU) made up of necrotic tissue debris and blood clot and diffuse leucocytic infiltration in the dermis (LI) at day 5 after burn, H&E ×100, (b) BW + 1% SSD, (h) BW + 5% ASHE, and (i) BW + 5% ASDE, all showing presence of epidermal epithelialization (E) with fibroblast proliferation (F) in the dermis at day 15 after burn, H&E ×400, (c) BW + 1% SSD, (d) BW + 2% ASHE, (e) BW + 2% ASDE, (f) BW + 1% ASHE, and (g) BW + 1% ASDE showing complete epidermal epithelialization (E) with presence of keratin proliferation, and fibroblast proliferation (F) in the dermis (D) at 20 days after burn and treatment, H&E ×400. Note the absence of exocrine glands and hair follicles in the dermis (D).

**Figure 5:**
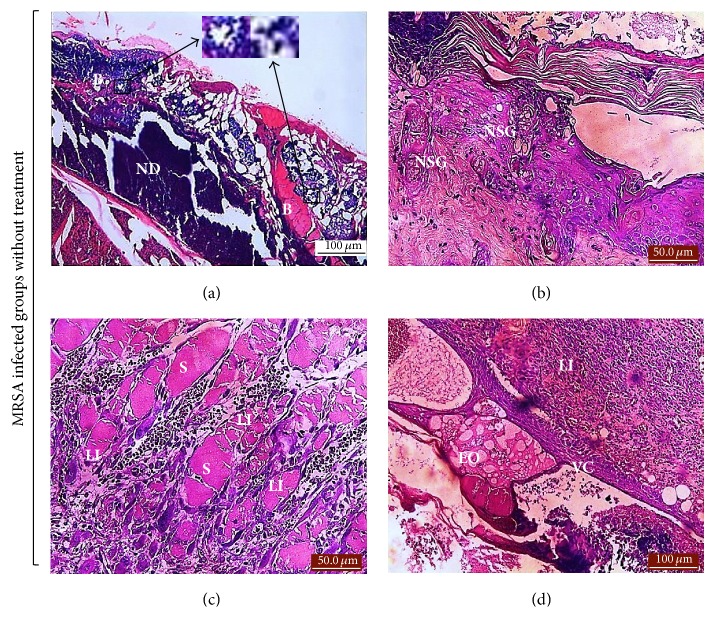
Representative photomicrographs of MRSA infected burn wound without treatment showing (a) large area of necrotic debris (ND) with multiple bacterial colonies in the dead tissue 10 days after infection with MRSA, H&E ×200. (b) A section of the skin showing necrotic sebaceous gland (NSG) in the dermis, characterized by pyknosis and karyolysis of the glandular cells, H&E ×400. (c) Multifocal areas of leucocytic infiltration (LI) around smooth muscle fibers (S) in the dermis, H&E ×200, (d) epidermal edema (EO), and vascular congestion (VC) with diffuse leucocytic infiltration (LI) in the dermis after 10 dpw, H&E ×200.

Histological scoring values of ASHE and ASDE treated groups along with controls are highlighted in Table 3. Congestion and edema were absent in both ASHE and ASDE treated groups. PMN and ulceration were observed in the untreated controls and were lower (*p* < 0.05) in the BW + ASDE (5%) group. However, MNC were observed in BW + SSD (1%), BW + MRSA + SSD (1%), and BW + ASDE (5%) groups, but lower (*p* < 0.05) than the untreated controls. Cellular degeneration and necrosis were absent in the drug treated controls, BW + MRSA + ASHE (1%), and BW + ASHE (2%). However, degeneration and necrosis were observed in other treated groups but were lower (*p* < 0.05) in BW + MRSA + ASDE (5%). Neovascularization was lower (*p* < 0.05) in the untreated control and higher (*p* < 0.05) in BW + SSD (1%) and both ASHE and ASDE treated groups. Both fibroblast proliferation and epidermal epithelialization were lower (*p* < 0.05) in the untreated control groups and higher (*p* < 0.05) in the treated control groups and both ASHE and ASDE treated groups. From the histopathological results, it is evident that treatment of mice burn with ASHE and ASDE did not show any visible bacterial cell aggregates in the tissues (after 5 dpw). Furthermore, complete epidermal reepithelialization and marked dermal fibroblast proliferation were observed after 15 dpw (Figure 4). On the other hand, MRSA infected tissues of control groups showed marked leucocytic infiltration, visible bacterial cells with cellular debris, epidermal edema, vascular congestion, and severe necrosis (Figure 5). Infected tissues were surrounded by the presence of cocci in clusters confirmed to be* S. aureus* colonization in the wound (Figure 5(a)).

### 3.6. Cytotoxicity Assay on Vero Cell Lines

**Figure 6:**
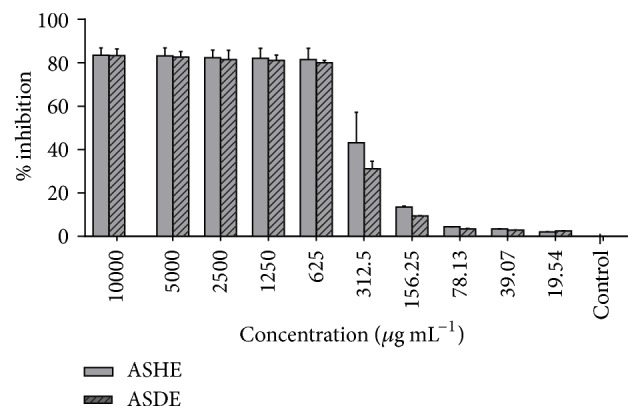
Effect of different concentrations of ASHE and ASDE on the viability of monkey kidney fibroblast cell line (Vero) after 24 h of incubation.

The cytotoxicity dose response readings after 24 h for ASHE and ASDE against Vero cells are shown in Figure 6. The concentrations of ASHE at 383.4 *μ*g mL^−1^ and ASDE at 390.6 *μ*g mL^−1^ still revealed more than 50% cell viability after 24 h of treatment.

## 4. Discussion

Bioactive compounds of* A. stipitatum* (Persian shallot) possess wound healing activity [16, 29]. The presence of fatty acids, linolenic acid (14.66%), and linoleic acid (78.88%) in* A. hirtifolium *bulbs has been reported earlier [30]. Significant amount of the presence of allicin (a major bioactive compound of* Allium* family) in the bulbs of* A. hirtifolium* Boiss. has also been demonstrated by TLC methods [31]. In contrast, only a low percentage of sulfur compounds including S-methyl methanethiosulfonate, 2,4,5-trithiahexane, 2,4-dithiapentane, 2-pyridinethione, and methane (chloromethylthio) (methylthio) were detected in the present study. Volatile sulfur compounds are known to be formed due to the enzymatic action of alliinase that cleaves* S*-alk(en)yl cysteine sulfoxide flavor precursors and lachrymatory-factor synthase enzymes. The presence of the aforementioned compounds in* A. stipitatum* has also been reported by other researchers [20]. Briefly, a recent study by Ismail et al. [20] on the GC-MS analysis of* A. hirtifolium* hydromethanolic extract showed the presence of 2-pyridinethione (1.6%). However, GC-MS analysis of ASHE and ASDE showed a higher concentration of 2-pyridinethione that ranged from 3.38 to 3.87%. In addition, both extracts also contain other bioactive sulfur compounds such as thiosulfuric acid (0.15–1.52%) and methanesulfonamide (5.23%) which were not reported in the hydromethanolic extract. The hydromethanolic extract of Persian shallot was effective against many bacterial pathogens including MRSA at 120 mg mL^−1^ (zone of inhibition: 18.17 ± 0.75 at 120 mg mL^−1^; MIC: 1.88 mg mL^−1^) [20]. However, the present study showed greater activity in the nonpolar extracts at low concentrations (10 mg mL^−1^ of extract exerted a zone of inhibition of 23–27 mm; MIC: 32–64 *μ*g mL^−1^) possibly due to the presence of high concentrations of volatile sulfur-containing compounds in ASHE and ASDE as compared to the hydromethanolic extract of Persian shallot.

*Staphylococcus aureus* is a predominant and challenging nosocomial pathogen of increasing importance. The lack of normal physical barrier associated with burn wounds allows such pathogens to colonize the exposed wound surface which is rich in fibronectin, fibrinogen, collagen, and so forth. In this study, MRSA was selected as a test microbe mainly due to its impressive resistance mechanisms towards commonly available antibiotics which remains a great challenge in burn wound care settings. Both ASHE and ASDE are proportionately strong antibacterials against MRSA. Analysis on the antibacterial activities of ASHE and ASDE based on the logarithmic reduction in bacterial CFUs suggests that the activity of ASHE and ASDE is much similar to SSD. ASHE (1%) and ASDE (1%) are also bactericidal with complete bacterial elimination being achieved after 96 hpi. The killing efficacy of SSD (positive control) against MRSA in mice burns was found to be 0.6 log_10_ reduction in CFU g^−1^ on the 4th day.

The antibacterial effects of ASHE and ASDE against MRSA was much similar with slight variation in the colony counts at 1% concentration (0.9 > 0.5 log_10_ reduction, *p* < 0.001). ASHE and ASDE at increasing concentrations exhibited greater activity against MRSA. At concentrations of 2% and 5%, complete antibacterial activity was achieved in 4 days suggesting that both extracts in ointment formulations possess strong antibacterial activities in a dose dependent manner. These results are partly in agreement with Nidadavolu et al. [32], who reported ~7 log_10_ reduction in* A. baumannii* and 8 log_10_ reduction in* S. aureus* biofilms after treatment with garlic oil for 32 h in vitro. No evidence of recurrence or regrowth of bacteria were observed in burn wounds treated with ASHE/ASDE after 4 dpw. CFU counts were zero in tissue samples collected on day 5 and day 6 suggesting a bactericidal activity by the two extracts.

Although, the present study used a different species of* Allium*, certain volatile organosulfur compounds are highly effective in controlling antibiotic resistant pathogens. The two extracts used in this study were in the crude form and formulated with SOB, and the variation in log_10_ reduction in bacterial CFUs could be due to the combination of biologically active compounds as observed by several other studies [16, 22, 33].

Chloroformic extract of garlic has been reported to be highly bactericidal against multidrug resistant strains of* Acinetobacter* sp. [34]. Nevertheless, the isolation of bioactive compounds in ASHE/ASDE and an ointment preparation with a single bioactive compound might have a much stronger activity as compared to the crude ointment formulations. Daily oral administration of garlic extract (50% and 100%), diallyl sulphides (5% and 10%), and diallyl disulphide (0.5% and 1%) and its diallyl sulphides in experimental rats was shown to protect mice against MRSA systemic infection [35]. It is evident from the above findings that hexane and dichloromethane extracts of Persian shallot (*A. stipitatum*) possess antibacterial properties against MRSA associated infections in thermal wounds.

Hydroalcoholic extract of* A. stipitatum* accelerated wound healing in rats with full-skin-thickness wounds by increasing the rate of epithelialization [16]. In this study, daily application of 5% ASHE and ASDE to MRSA infected and uninfected mice with burn wounds showed significant reduction in wound size (*p* < 0.001), with complete epithelialization and wound contraction in ~13–15 days as compared to the controls (>20 days). Alcoholic extract of* A. sativum* has been reported to possess antibacterial and burn wound healing properties in a rabbit model of burn wound resulting in complete burn wound healing after daily application for 21 days [33]. In this study, ASHE and ASDE formulated ointments adequately aided in tissue remodeling and increased burn wound healing, which is noteworthy for a natural extract. The histopathological lesions were comparatively different in ASHE/ASDE treated and untreated groups. Evidence of congestion, edema, polymorpho, and mononuclear infiltrates and ulceration are absent in ASHE/ASDE treated groups as compared to the controls. Increasing concentrations of the extract formulated ointments showed an increased rate of fibroblast proliferation and epidermal epithelialization. After 20 dpw, ASHE and ASDE at 1% concentrations were still effective. However, ASHE and ASDE (2% and 5%) showed a relatively enhanced wound healing through antibacterial activity and advanced collagen deposition. Despite the antibacterial and wound healing properties, ASHE and ASDE ointment formulations applied to mice burn wounds formed a pseudoeschar on the wound surface promoting its bioavailability. This pseudoeschar cohering intrusively to mice tissues could be one of the main reasons for ASHE/ASDE to remain on burn wounds [23]. No such pseudoeschar is formed in mice groups treated with SOB or SSD ointment.

The cytotoxicity study suggests that hexane and dichloromethane extracts of* A. stipitatum* are nontoxic to Vero cells at <400 *μ*g mL^−1^. The MIC/MBCs of ASHE (32/128 *μ*g mL^−1^) and ASDE (64/128 *μ*g mL^−1^) for MRSA are comparatively lesser than the CC_50_ concentrations on Vero cells (<383.4 *μ*g mL^−1^; <390.6 *μ*g mL^−1^) which is therapeutically acceptable for a nontoxic edible natural extract. Chloroformic extract of* A. hirtifolium* bulbs suppressed the growth of HeLa and MCR-7 cells at 200 *μ*g mL^−1^ [31], while the hydromethanolic extract of* A. hirtifolium* remains nontoxic to mammalian cells at 1500 *μ*g mL^−1^ [20]. These results are partly in agreement with our results, which for the first time report the in vivo antibacterial and burn wound healing activities of hexane and dichloromethane extracts prepared from the bulbs of* A. stipitatum*.

## 5. Conclusion

In conclusion, the present investigation demonstrated the in vivo antibacterial and burn wound healing potential of* A. stipitatum* extract formulated ointments. Both extracts (in crude form and ointment formulation) are equally effective against MRSA in mice burns. Complete removal of burn pathogens from burn wounds was achieved at 48–72 hpi and treatment. ASHE and ASDE ointment formulations uniformly accelerated burn wound healing by increasing the rate of new blood vessel formation, fibroblast proliferation, and epithelialization of the epidermis. The rate of wound contraction was higher (95–100%) in mice groups treated with ASHE/ASDE formulated ointments after 15 dpw. ASHE and ASDE are noncytotoxic and hence could be safe for mammalian cells (383.4 *μ*g mL^−1^; 390 *μ*g mL^−1^), which suggests that the extracts could be used as a safe topical antibacterial and a promising alternative in the ambience of MRSA burn wound infections.

## Supplementary Material

Effect of SOB and SSD on burn wound contraction in MRSA infected and uninfected control groups. Representative images of burn wound healing in (A) MRSA infected and uninfected groups treated with SOB and SSD; (B) MRSA infected and uninfected groups treated with ASHE (1%, 2% & 5%); (C) MRSA infected and uninfected groups treated with ASDE (1%, 2% & 5%) on different dpw (5 days interval). Abbreviations: BW - burn wound; SOB - simple ointment base; MRSA - methicillin-resistant S. aureus; SSD - silver sulfadiazine; G - Group; ASHE - Allium stipitatum hexane extract; ASDE - Allium stipitatum dichloromethane extract.

## Figures and Tables

**(a):** 

Serial number	Peak RT (min)	Peak area (%)	Compound detected	Major group	CAS number	Mol. formula	Mol. wt.
1	4.472	0.94	Pentanone,4-hydroxy-4-methyl	Synthetic intermediate	123-43-2	C_5_H_10_O	86.1323

2	11.561	2.11	1,2,4-Trimethylbenzene	Aromatic hydrocarbon	95-63-6	C_9_H_12_	120.19

3	21.586	10.11	Citronellal	Monoterpenoid	106-23-0	C_10_H_18_O	154.25
4	31.599	2.26	Citronellol isobutanoate	Aromatic ether	97-89-2	C_14_H_26_O_2_	226.36
5	32.338	3.87	2-Pyridinethione	Organic sulfur compound	2637-34-5	HSC_5_H_4_N	111.16

6	55.638	16.36	Hexadecanoic aci d	Aliphatic acids	57-10-3	C_16_H_32_O_2_	256.42

7	57.858	1.52	Thiosulfuric acid	Sulfur oxoacid	2937-53-3	H_2_S_2_O_3_	114.14
8	61.176	19.17	9,12-Octadecadienoic acid	Essential fatty acids	60-33-3	C_18_H_32_O_2_	280.45

9	61.714	4.69	Benzenemethanol<4-hydroxy->	Phenolic alcohols	623-05-2	C_7_H_8_O_2_	124.1372
10	65.348	19.37	Ipsdienol	Cyclic esters	35628-00-3	C_10_H_16_O	152.23
			Hexalactone<gamma->		695-06-7	C_6_H_10_O_2_	114.144
			Decalactone<gamma->		706-14-9	C_10_H_18_O_2_	170.25

11	67.053	1.10	No matches found	—	—	—	—
12	41.47	0.31	Unknown	—	—	—	—

**(b):** 

Serial number	Peak RT (min)	Peak area (%)	Compound detected	Major group	CAS number	Mol. formula	Mol. wt.
1	3.339	0.89	Pentyl propanoate	Organic ester	123-42-2	C_8_H_16_O_2_	144.22
			Propanoic acid hexyl ester	Fatty acids	2445-76-3		

2	3.425	2.41	1-Hexen-3-ol<1->	Aromatic alcohol	4798-44-1	C_6_H_12_O	100.16
3	3.590	1.61	Butanediol<2,3->	Organic glycol	513-85-9	C_4_H_10_O_2_	90.121
4	4.477	1.06	Pentanone<4-hydroxy-4-methyl-2->	Synthetic intermediate	123-42-2	C_6_H_12_O_2_	116.16

5	21.597	11.32	Citronellal	Monoterpenoid	106-23-0	C_10_H_18_O	154.25
6	31.605	2.35	Citronellyl isobutanoate	Sulfonates	97-89-2	C_14_H_26_O_2_	226.36
7	32.361	3.38	2-Pyridinethione	Organosulfur compound	2637-34-5	HSC_5_H_4_N	111.16

8	48.187	1.68	2-Pyridinethione	Organosulfur compound	2637-34-5	HSC_5_H_4_N	111.16

9	55.683	15.96	Decanoic acid	Aliphatic acids	334-48-5	C_10_H_20_O_2_	172.26
			Dodecanoic acid		143-07-7	C_25_H_52_O_8_	480.67

10	55.706	7.81	Hexadecanoic acid	Saturated fatty acid	57-10-3	C_16_H_32_O_2_	256.42

11	56.707	0.15	Thiosulfuric acid	Sulfur oxoacid	2937-53-3	H_2_S_2_O_3_	114.14
12	56.759	0.25	Cysteaminesulfonic acid	Sulfur compounds	54641-86-0	C_2_H_7_NO_3_S_2_	157.21
			S-beta- Aminoethylthiosulfuric acid		2937-53-3	C_2_H_8_NO_3_S_2_	158.32

13	56.759	1.49	1-Formyl-2,2,6-trimethyl-3-(3-methyl-2-buten-1-yl)-6-cyclohexene	—	8287-15-6	C_15_H_24_O	220.350

14	60.049	2.69	Furfuryl butanoate	Aromatic compound	623-21-2	C_9_H_12_O_3_	168.18
15	61.125	14.88	9,12-Octadecadienoic acid	Polyunsaturated fatty acid	2197-37-7	C_18_H_32_O_2_	280.44
16	61.142	7.42	Linoleic acid	Polyunsaturated fatty acid	60-33-3	C_18_H_32_O_2_	280.44

17	62.035	0.36	9-Octadecanoic acid	Polyunsaturated fatty acid	112-79-8	C_18_H_34_O_2_	282.46
18	63.225	5.23	Methanesulfonamide	Sulfonamides (sulfa drugs)	3144-09-0	CH_5_NO_2_S	95.12

**Table 2:** Zone of inhibition, MIC, and MBC of ASHE and ASDE against MRSA (20 *μ*L corresponding to 200 *μ*g/disc).

Strain	Zone of inhibition (mm)				MIC (*μ*g mL^−1^)		MBC (*μ*g mL^−1^)	
	ASHE	ASDE	Antibiotic	DMSO (10%)	ASHE	ASDE	ASHE	ASDE
MRSA ATCC 43300	27 ± 0.5774	23 ± 0.2887	16 (van)	—	32	64	128	128

van: vancomycin (30 *μ*g)

— means no zone of inhibition.

**Table 3:** Histological lesion score in mice skin tissue with various treatments examined at day 20.

Group/treatment	C	ED	PMN	MNC	U	D & N	NV	FP	EP
BW only	3.0 ± 0.0	1.7 ± 0.21	3.0^a^ ± 0.0	3.0^a^ ± 0.0	2.2^a^ ± 0.16	1.8^a^ ± 0.16	0.5^a^ ± 0.22	0.5^a^ ± 0.2	1.7^a^ ± 0.21
BW + SOB	2.5 ± 0.22	2.2 ± 0.16	2.3^b^ ± 0.21	2.0^b^ ± 0.0	1.0^b^ ± 0.0	1.7^a^ ± 0.21	1.5^b^ ± 0.22	0.7^a^ ± 0.21	1.2^a^ ± 0.16
BW + MRSA	3.0 ± 0.0	1.8 ± 0.16	3.0^a^ ± 0.0	3.0^a^ ± 0.0	2.2^a^ ± 0.16	2.0^a^ ± 0.0	0.7^a^ ± 0.21	0.7^a^ ± 0.21	0.8^b^ ± 0.16
BW + MRSA + SOB	3.0 ± 0.0	1.7 ± 0.21	2.5^b^ ± 0.22	2.5^c^ ± 0.22	2.0^a^ ± 0.0	1.8^a^ ± 0.16	0.7^a^ ± 0.21	1.0^a^ ± 0.0	1.2^a^ ± 0.16
BW + SSD (1%)	0	0	0	0.3^d^ ± 0.21	0	0	2.5^c^ ± 0.22	3.0^b^ ± 0.0	3.0^c^ ± 0.0
BW + MRSA + SSD (1%)	0	0	0	0.5^d^ ± 0.22	0	0	0.7^a^ ± 0.21	3.0^b^ ± 0.0	3.0^c^ ± 0.0

BW + MRSA + ASHE (1%)	0	0	0	0	0	0	2.5^c^ ± 0.22	2.0^c^ ± 0.0	2.5^c^ ± 0.22
BW + MRSA + ASHE (2%)	0	0	0	0	0	1.5^a^ ± 0.22	2.3^c^ ± 0.21	2.5^b^ ± 0.22	3.0^c^ ± 0.0
BW + MRSA + ASHE (5%)	0	0	0	0	0	1.7^a^ ± 0.21	2.5^c^ ± 0.22	2.5^b^ ± 0.22	3.0^c^ ± 0.0
BW + ASHE (1%)	0	0	0	0	0	2.3^ab^ ± 0.21	2.7^c^ ± 0.21	3.0^b^ ± 0.0	3.0^c^ ± 0.0
BW + ASHE (2%)	0	0	0	0	0	0	2.5^c^ ± 0.22	3.0^b^ ± 0.0	3.0^c^ ± 0.0
BW + ASHE (5%)	0	0	0	0	0	2.5^ab^ ± 0.22	3.0^c^ ± 0.0	3.0^b^ ± 0.0	3.0^c^ ± 0.0

BW + MRSA + ASDE (1%)	0	0	0	0	0	1.7^a^ ± 0.21	1.7^c^ ± 0.21	2.5^b^ ± 0.22	2.5^c^ ± 0.22
BW + MRSA + ASDE (2%)	0	0	0	0	0	1.7^a^ ± 0.21	2.3^c^ ± 0.21	2.5^b^ ± 0.22	3.0^c^ ± 0.0
BW + MRSA + ASDE (5%)	0	0	0	0	0	1.0^c^ ± 0.0	2.7^c^ ± 0.21	2.5^b^ ± 0.22	3.0^c^ ± 0.0
BW + ASDE (1%)	0	0	0	0	0	2.0^a^ ± 0.0	2.5^c^ ± 0.22	3.0^b^ ± 0.0	3.0^c^ ± 0.0
BW + ASDE (2%)	0	0	0	0	0	2.2^a^ ± 0.16	2.5^c^ ± 0.22	2.5^b^ ± 0.22	3.0^c^ ± 0.0
BW + ASDE (5%)	0	0	1.5^c^ ± 0.22	0.5^d^ ± 0.22	0.2^c^ ± 0.16	2.3^ab^ ± 0.16	2.5^c^ ± 0.22	3.0^b^ ± 0.0	3.0^c^ ± 0.0

^a,b,c,d^Superscripts with different letters in a column are significantly different at *p* > 0.05. Values are expressed as mean ± SEM (0: no lesion distribution; 1: mild distribution; 2: moderate distribution; and 3: marked distribution).

BW: burn wound; SOB: simple ointment base; MRSA: methicillin-resistant *S. aureus*; SSD: silver sulfadiazine; ASHE: *A. stipitatum* hexane extract; ASDE: *A. stipitatum* dichloromethane extract; C: congestion; ED: edema; PMN: polymorphonuclear cells; MNC: mononuclear cells; U: ulceration; D & N: degeneration and necrosis; NV: neovascularization; FP: fibroblast proliferation; EP: epithelialization. Values are presented as the average of one unit square, that is, 2 × 2 mm^2^ area of tissue with lesions.
