# Supplementary material for: Allium stipitatum Extract Exhibits In Vivo Antibacterial Activity against Methicillin-Resistant Staphylococcus aureus and Accelerates Burn Wound Healing in a Full-Thickness Murine Burn Model
Source: Evid Based Complement Alternat Med. 2017 Feb 22;2017:1914732. doi: 10.1155/2017/1914732 (PMC5340985; doi:10.1155/2017/1914732)
Supplement: Supplementary file 1 — Effect of SOB and SSD on burn wound contraction in MRSA infected and uninfected control groups. Representative images of burn wound healing in (A) MRSA infected and uninfected groups treated with SOB and SSD; (B) MRSA infected and uninfected groups treated with ASHE (1%, 2% & 5%); (C) MRSA infected and uninfected groups treated with ASDE (1%, 2% & 5%) on different dpw (5 days interval). Abbreviations: BW - burn wound; SOB - simple ointment base; MRSA - methicillin-resistant S. aureus; SSD - silver sulfadiazine; G - Group; ASHE - Allium stipitatum hexane extract; ASDE - Allium stipitatum dichloromethane extract. [file 1914732.f1.docx]

**A**


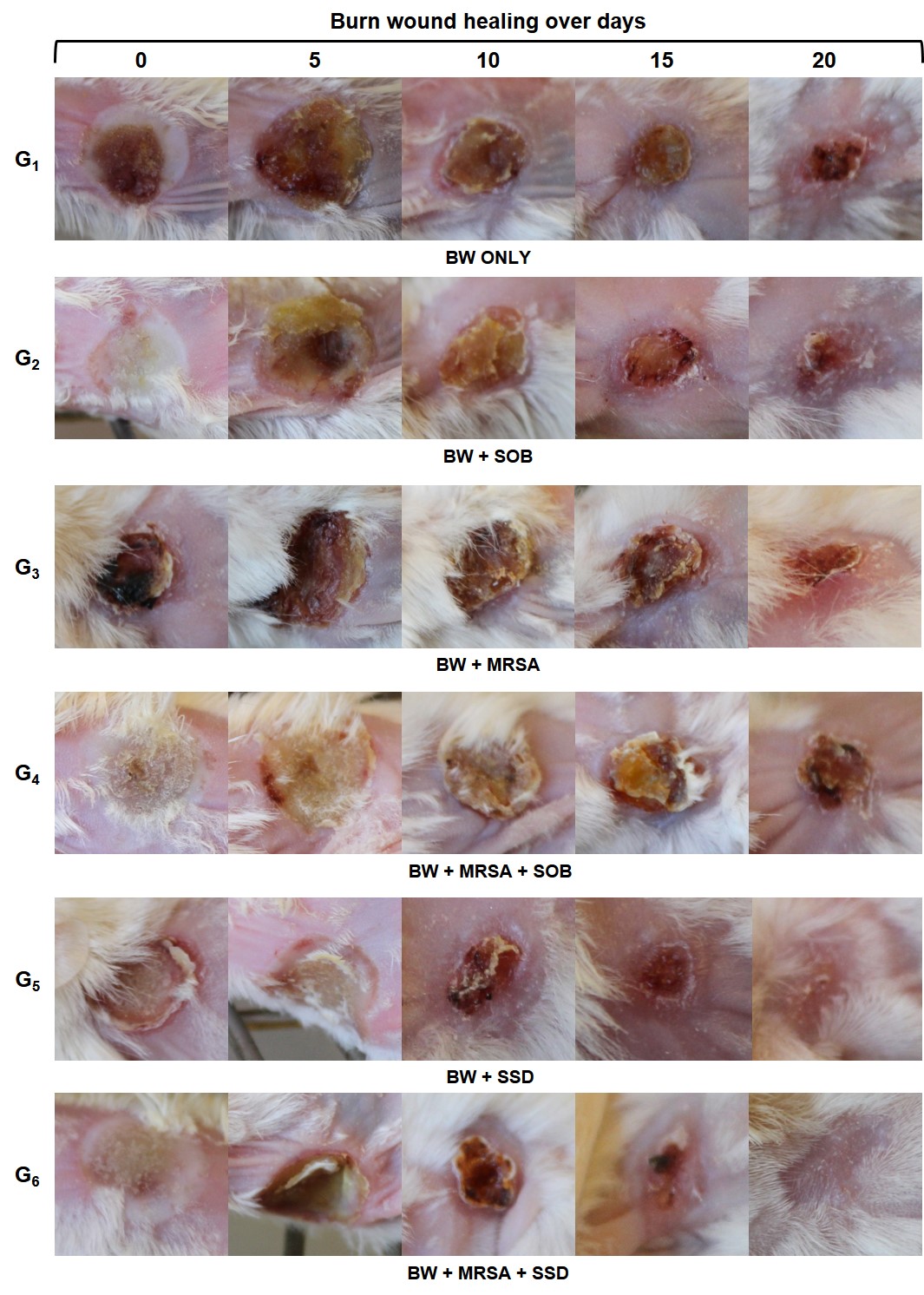


**B**


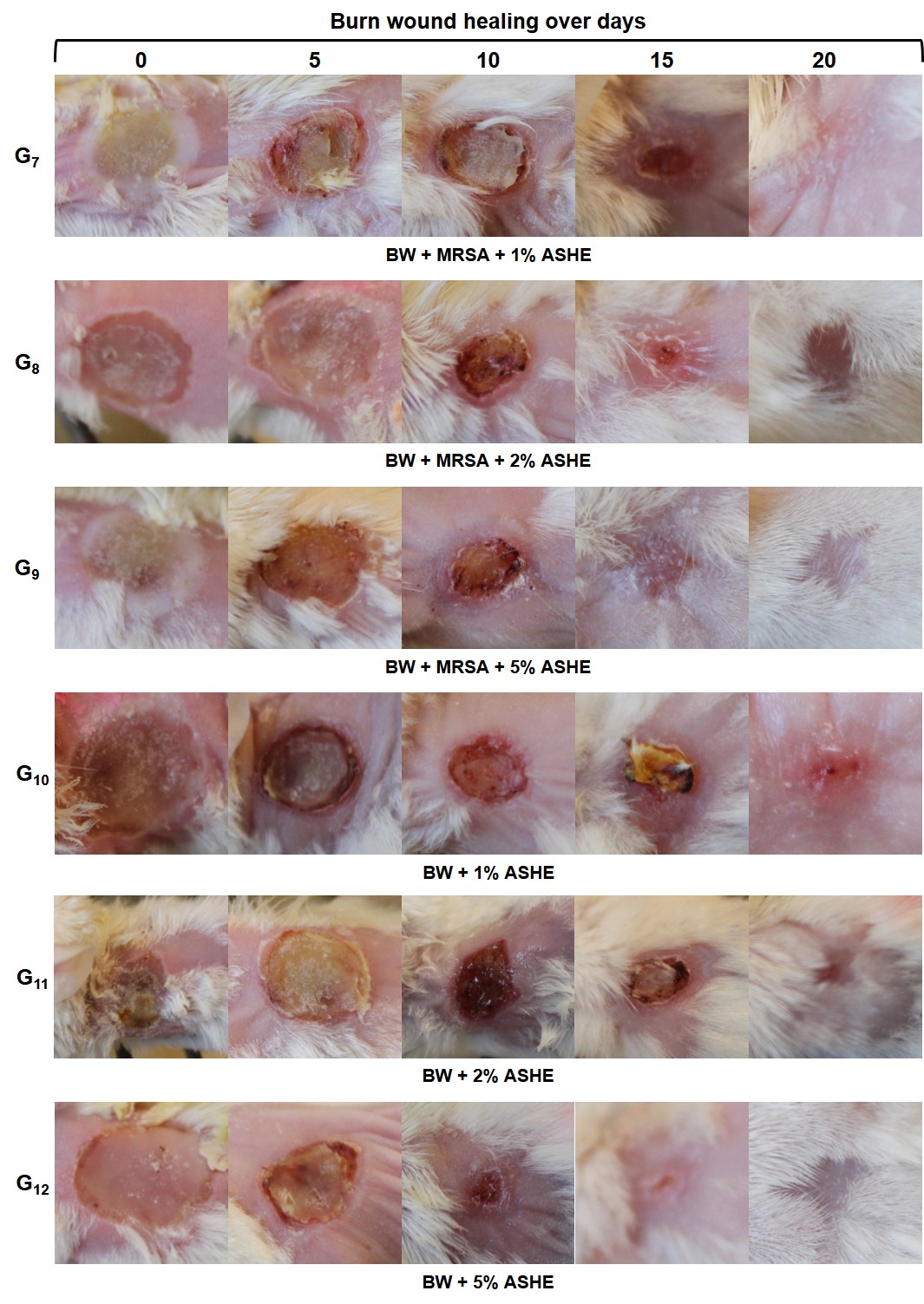


**C**


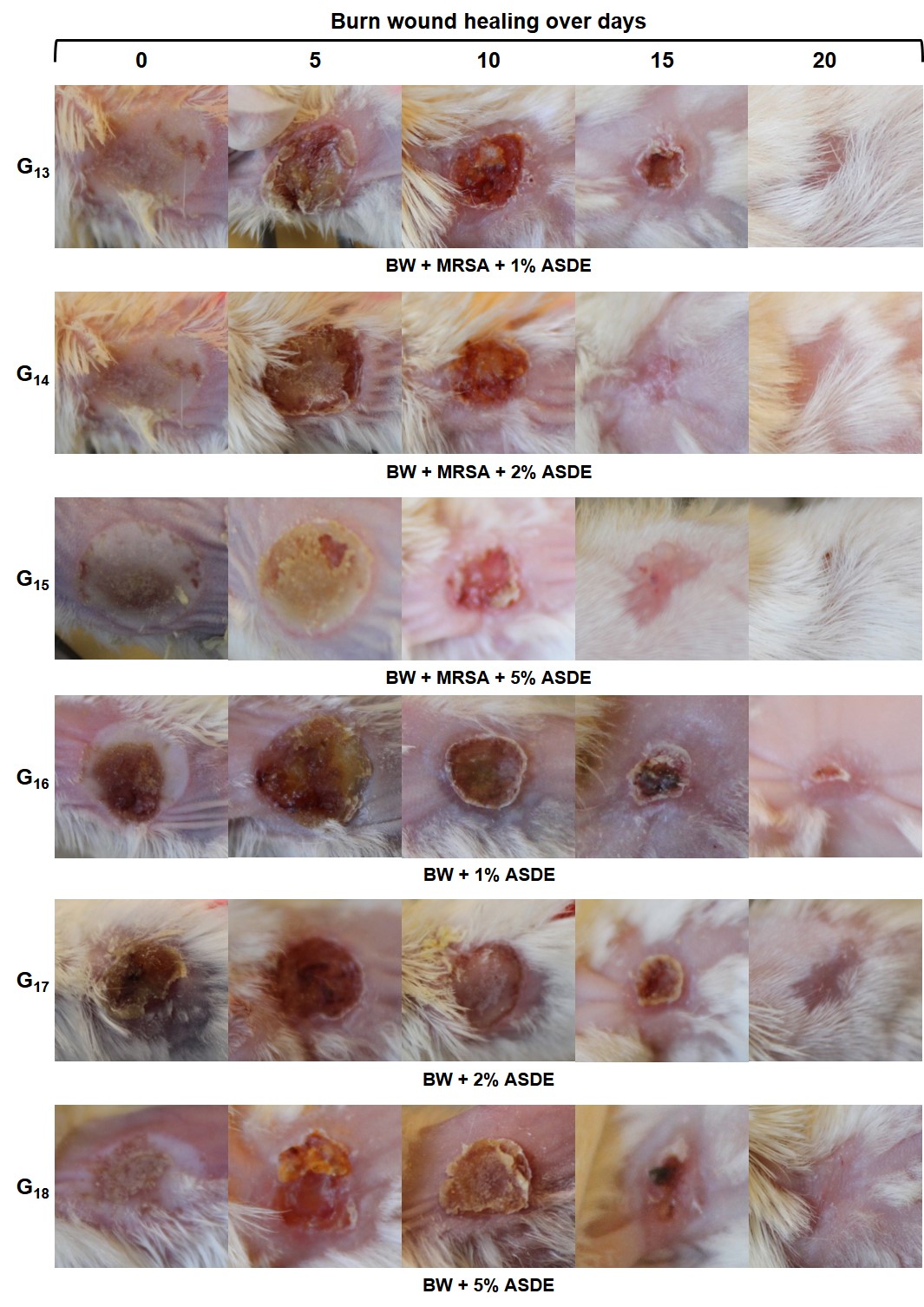


FIGURE S1: Effect of SOB and SSD on burn wound contraction in MRSA infected and uninfected control groups. Representative images of burn wound healing in (**A**) MRSA infected and uninfected groups treated with SOB and SSD; (**B**) MRSA infected and uninfected groups treated with ASHE (1%, 2% & 5%); (**C**) MRSA infected and uninfected groups treated with ASDE (1%, 2% & 5%) on different dpw (5 days interval).

**Abbreviations: BW -** burn wound; **SOB -** simple ointment base; **MRSA -** methicillin-resistant *S. aureus*; **SSD -** silver sulfadiazine; **G** - Group; **ASHE -** *Allium stipitatum* hexane extract; **ASDE** - *Allium stipitatum* dichloromethane extract.
